# Full-Mouth Disinfection Using Oral Sitafloxacin for Stage III and IV Grade C Periodontitis With High Bacterial Load: A Case Series

**DOI:** 10.7759/cureus.78531

**Published:** 2025-02-05

**Authors:** Satoru Morikawa, Takazumi Yasui, Taneaki Nakagawa

**Affiliations:** 1 Department of Dentistry and Oral Surgery, Keio University School of Medicine, Tokyo, JPN

**Keywords:** bacterial load, debridement, periodontitis, root planing, sitafloxacin

## Abstract

This case series presents a novel treatment approach for severe periodontitis with high bacterial loads in four patients (aged 32-47 years), including three cases of stage III and one case of stage IV, grade C periodontitis. After conventional scaling and root planing failed in one case, we developed a protocol combining full-mouth disinfection (FMD) with oral sitafloxacin (STFX) for cases with high levels of subgingival periodontal pathogens. FMD involved thorough debridement within 1 week, with STFX (100 mg/day) administered for 7 days. Clinical and microbiological parameters were evaluated before and after treatment. The results showed marked improvements in probing pocket depth, bleeding on probing, and inflamed periodontal surface area, with substantial reductions in red complex bacteria. This approach often eliminates the need for periodontal surgery, even in deep pockets, suggesting FMD with STFX as an effective nonsurgical alternative for severe periodontitis with significant bacterial load.

## Introduction

Periodontitis is a highly prevalent disease characterized by the progressive loss of periodontal tissues, and untreated periodontitis can even result in tooth loss. Scaling and root planing (SRP), a mechanical debridement method, is considered the basic treatment to eliminate the infection source and periodontal pathogens from periodontal pockets. Previous research showed that the translocation of bacteria from untreated to treated areas following conventional SRP causes reinfection [1].

In the early 1990s, the concept of the sequential quadrant or sextant-wise SRP administration over 1-2 weeks was re-examined, and full-mouth disinfection (FMD) was introduced. Quirynen et al. [2] suggested that one-stage FMD involving complete SRP of both jaws within 24 h and adjunctive use of chlorhexidine could help prevent bacterial reinfection and reduce treatment frequency. Despite these benefits, FMD can cause systemic inflammatory symptoms, including an increased body temperature, shortly after therapy [3]. Additionally, FMD can cause temporary hypercytokinemia because of the spread of inflammatory mediators from periodontal tissues and pockets to other areas throughout the body [3]. However, undergoing FMD with antimicrobial administration reportedly improves the clinical parameters and lowers periodontal bacterial levels and body temperature [4]. Furthermore, based on our reflections on a case, FMD and sitafloxacin were introduced to treat generalized stage IV, grade C periodontitis [5] (particularly for patients with high bacterial levels), in which conventional quadrant-mouth SRP was performed with no improvement in the clinical parameters. In this case, FMD resulted in improved clinical and bacteriological findings, including those of quantitative polymerase chain reaction (qPCR).

Oral antimicrobial therapy should be utilized as an adjuvant to the primary periodontal treatment, as periodontal bacteria can be suppressed using the new broad-spectrum antimicrobial agents. Antimicrobial agents are used because they effectively suppress periodontal bacteria; new broad-spectrum antimicrobial agents, such as quinolones, are frequently employed in periodontitis cases. Sitafloxacin, a novel oral fluoroquinolone with broad-spectrum activity, is effective against bacteria with both Gram-positive- and -negative phenotypes, anaerobic bacteria, unusual pathogens, and bacteria resistant to other fluoroquinolones [6]. The cis-fluorocyclopropylamine group found in sitafloxacin has strong pharmacokinetic characteristics and can potentially alleviate cytotoxicity-related side effects. Sitafloxacin is primarily used to treat respiratory infections and severe refractory infectious diseases. Furthermore, in this study, clinical metrics such as probing pocket depth (PPD), bleeding on probing (BOP), and tooth mobility showed improvements following FMD and sitafloxacin administration. Our hypothesis, based on literature is that the use of FMD and sitafloxacin would be effective against severe periodontitis with substantial levels of bacterial factors. Here, we intend to assess the level of periodontal bacteria detected via qPCR before and after FMD and sitafloxacin treatment in four patients and propose an antimicrobial treatment protocol to reinforce the clinical and microbiological effectiveness of FMD.

In the original FMD method, debridement of the entire dentition is completed using 0.2-1% chlorhexidine within 24 h. However, since the subgingival use of 0.2-1% chlorhexidine is not approved in Japan, and because of patient weariness in the dental chair, we defined “FMD” as full-mouth SRP with complete debridement of the entire dentition within 2 days to 1 week. The purpose of this study was to evaluate the performance of the suggested therapeutic strategy in patients with severe periodontitis and high bacterial loads, including three cases of generalized stage III, grade C periodontitis, and one case of generalized stage IV, grade C periodontitis.

## Case presentation

The study was conducted in accordance with the guidelines of the Declaration of Helsinki and approved by the Ethics Committee of Keio University (approval number: 20210002, approval date: May 6, 2021). Written informed consent was obtained from all participants for the inclusion of their clinical data in this case series. Furthermore, all patients explicitly agreed to undergo the proposed FMD treatment as part of their periodontal therapy.

Case 1: Non-responsive to conventional scaling and root planing (SRP) treatment

**Figure 1:**
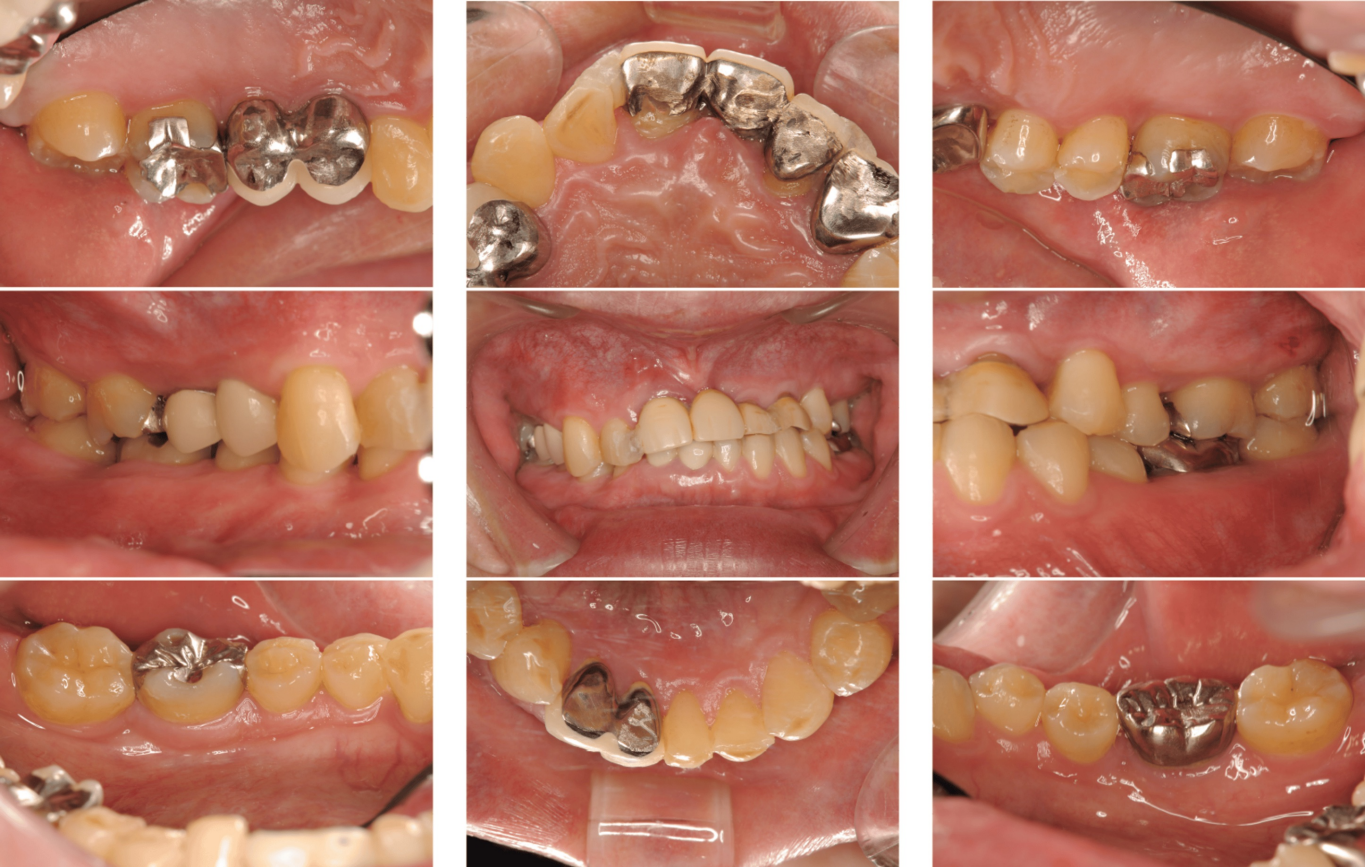
Photographs of a patient diagnosed with generalized stage III, grade C periodontitis (Case 1). Clinical examination showed signs of periodontal inflammation and deep periodontal pockets.

A 39-year-old Japanese man presented to our department with complaints of gingival swelling, pain, and tooth mobility; he had no history of systemic diseases. An initial examination revealed poor oral hygiene with a high plaque score, and a substantial decrease in the occlusal vertical dimension, disruption of the occlusal plane, and wear of multiple prostheses (Figure 1).

**Figure 2:**
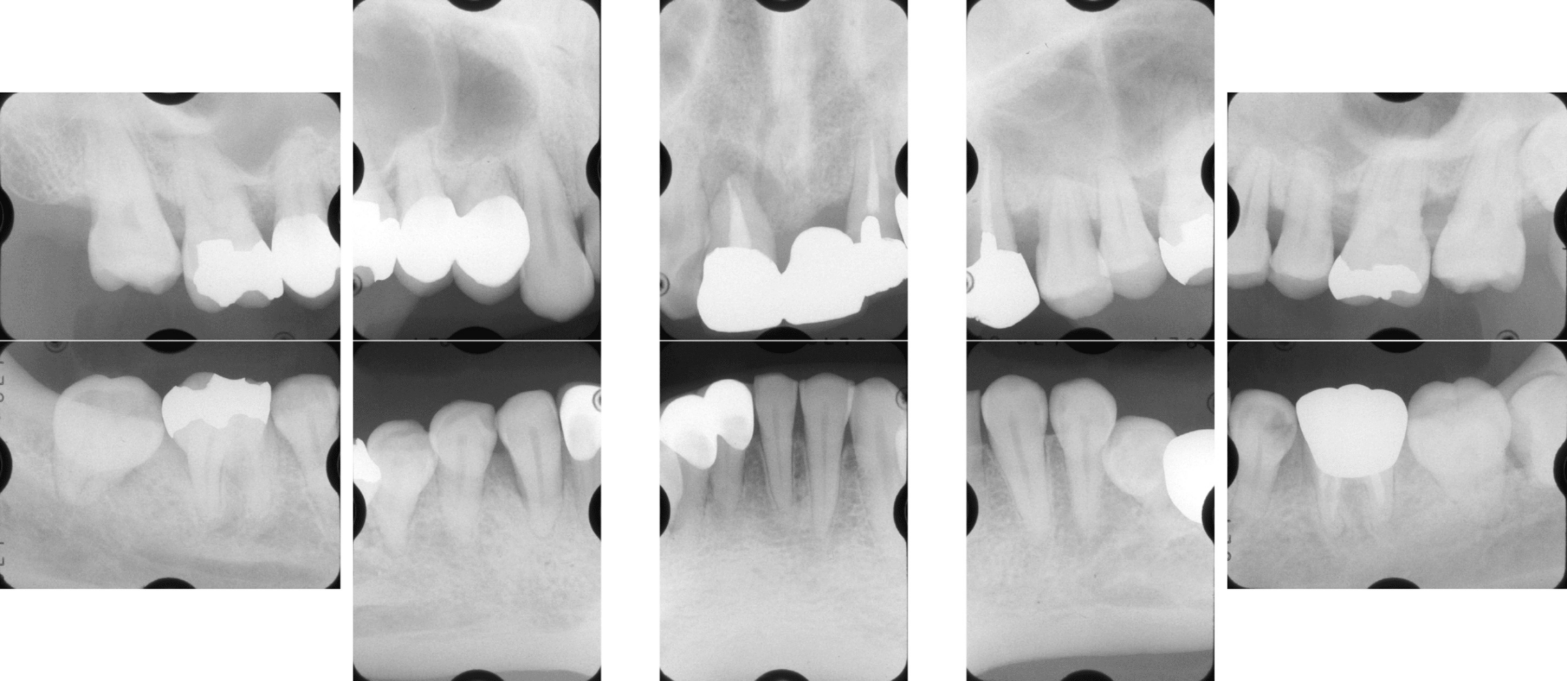
Baseline radiographs of a patient with generalized stage III, grade C periodontitis. Vertical and horizontal bone loss, including bone resorption at the root apex, was observed.

Clinical periodontal examination revealed that sites with PPD ≥ 7 mm comprised 28% of all measured sites. Similarly, radiographic examination revealed angular bone resorption on the mesial aspect of tooth #17 (Fédération Dentaire Internationale System), the distal aspect of tooth 15, and the distal aspect of tooth 26, with generalized horizontal alveolar bone resorption and bone resorption at the root apex of tooth 11 (Figure 2).

**Figure 3:**
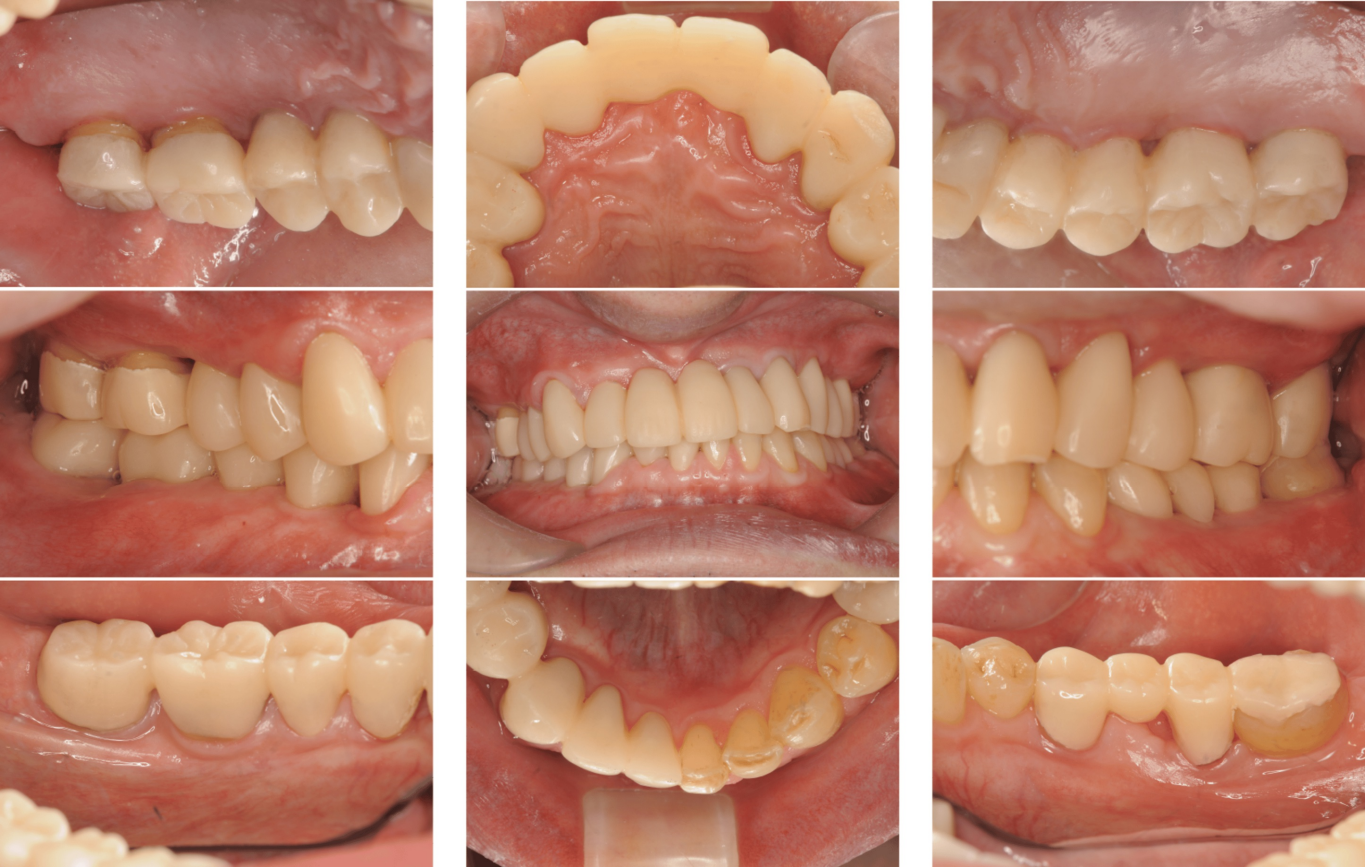
Photographs after quadrant-wise SRP with multiple appointments over 1-2 weeks of a patient with localized stage III, grade C periodontitis (Case 1). Despite quadrant-wise SRP over multiple appointments, clinical improvement was limited. SRP: scaling and root planing

**Figure 4:**
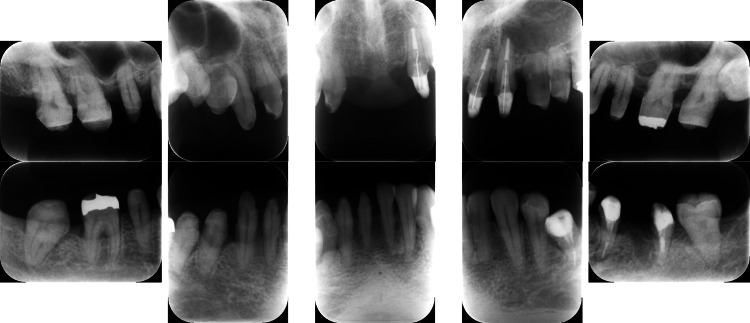
One-year follow-up radiographs after quadrant-wise SRP in a patient with localized stage III, grade C periodontitis (Case 1). No significant bone regeneration was observed after quadrant-wise SRP. SRP: scaling and root planing

We provided thorough instructions on the maintenance of oral hygiene. A tooth with a hopeless prognosis (#11) and a mandibular third molar (#38) were extracted, and SRP was performed quadrant-wise with multiple appointments spread over 1-2 weeks (Figures 3-4).

This method has been found to perform adequately as a conventional basic periodontal treatment. After the SRP procedure, approximately 1 year was necessary to reevaluate the patient because of the need for root canal treatments of multiple teeth, removal of a maxillary cyst, placement of a temporary crown to correct the occlusal height, and repair of the temporary crown fracture while continuing basic periodontal treatment, such as brushing and scaling procedures.

However, reevaluation after conventional basic periodontal treatment showed no improvement in the clinical parameters, including PPD (Table 1).

**Table 1:** Clinical characteristics at baseline and after FMD and STFX protocol BOP: bleeding on probing; FMD: full-mouth disinfection; PPD: probing pocket depth; PISA: periodontal inflamed surface area; SRP: scaling and root planing; STFX: sitafloxacin

	Case 1		Case 2		Case 3		Case 4	
Clinical parameters	Baseline	SRP	Baseline	FMD and STFX	Baseline	FMD and STFX	Baseline	FMD and STFX
BOP (mean %)	53.7	66.0	74.7	21.0	95.0	13.2	67.3	33.3
PPD ≥ 6 mm (mean %)	7.4	7.7	29.3	1.2	33.9	0.0	26.3	3.3
PPD (mm; mean)	3.3	3.6	4.7	2.6	5.1	2.3	4.7	2.5
PISA (mm^2^)	1131.3	1470.2	2436.7	296.5	2706.3	164.8	2547.7	378.9

The periodontal inflamed surface area (PISA), an index measuring the periodontal pocket inflammatory area based on the clinical attachment level, BOP, and the extent of gingival recession [7], can reveal the severity of periodontitis and the extent of the inflammatory wound. The PISA increased from 1131.3 mm^2^ to 1470.2 mm^2^ (Table 1). Since successful periodontal treatment reduces PPD and decreases the levels of harmful bacteria, including red complex bacteria and other periodontopathogens [8], in the periodontal pockets, we suspected the presence of residual red complex bacteria in the periodontal pockets. After reevaluation, qPCR showed persistent red complex bacteria (Table 2), probably because of reinfection of the treated sites due to bacterial translocation from untreated sites between SRP sessions.

**Table 2:** Quantitative evaluation of red complex bacteria in the deepest periodontal pocket after Case 1 SRP SRP: scaling and root planing

Subgingival bacteria	Counts in Case 1 after SRP
Total bacteria	490,000
Porphyromonas gingivalis	<10
Tannerella forsythia	11,000
Treponema denticola	2500

Based on the clinical experience and investigation findings, FMD was scheduled to complete SRP of all teeth within 1 week to prevent reinfection. Additionally, we administered sitafloxacin (days 1 to 7, dose: 100 mg) and observed a reduction in the bacterial load and improved clinical parameters (data not shown). Moreover, this protocol was used for Cases 2, 3, and 4, in which three patients (all women; age range, 32-47 (mean age, 40) years) were selected from among those who visited our department between 2012 and 2017. The patients included three cases of generalized stage III, grade C periodontitis (Cases 1, 2, and 3) and one case of generalized stage IV, grade C periodontitis (Case 4) [5].

Case 2

**Figure 5:**
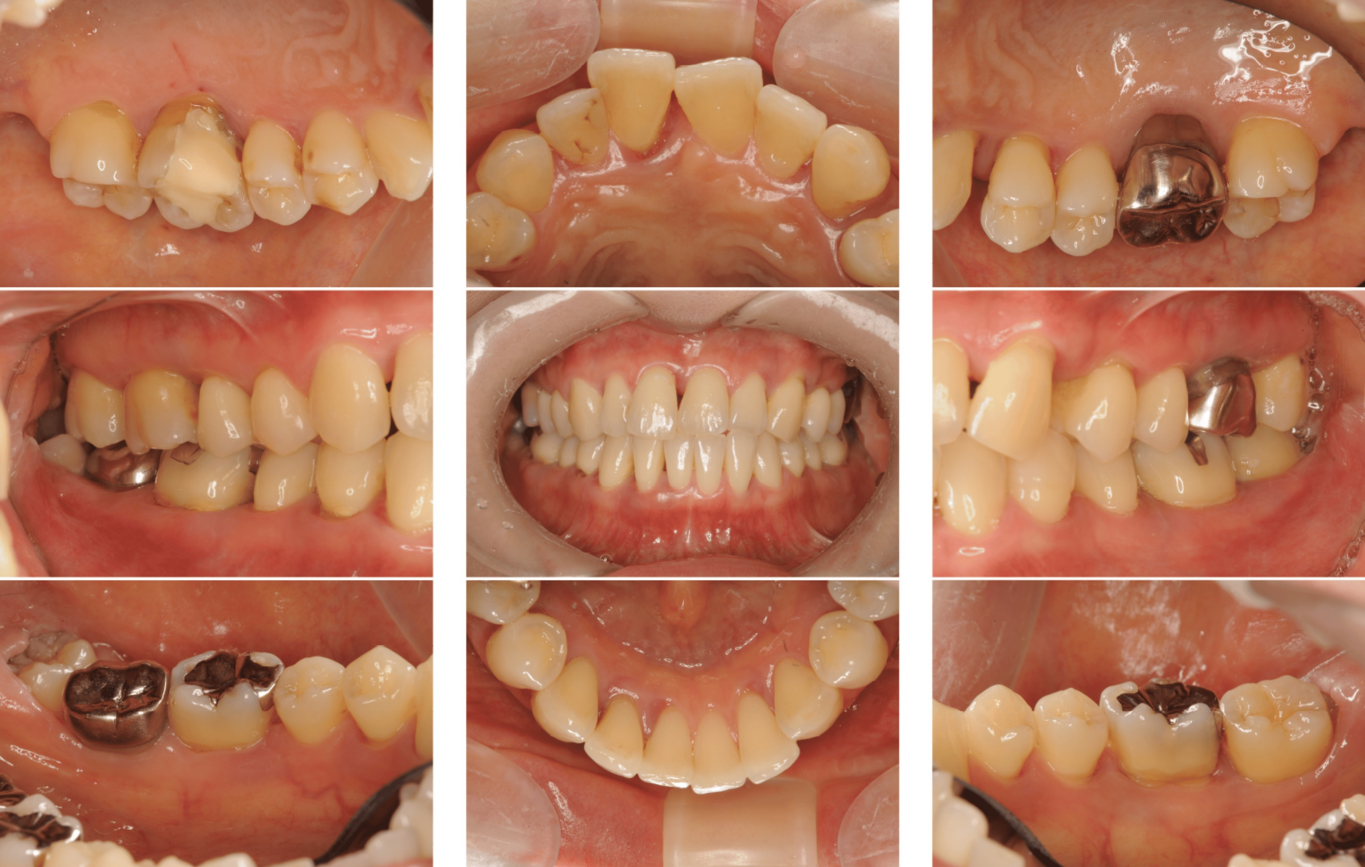
Baseline clinical photographs of a patient with generalized stage III, grade C periodontitis (Case 2). The patient exhibited periodontal inflammation with deep probing pocket depths.

A 32-year-old Japanese woman with severe periodontitis was referred to our department for complete assessment and treatment. The patient had no medical history. Since her early twenties, she had been aware of occasional gingival swelling and pain; however, no active treatment for periodontal disease had been offered at her local dental clinic. During the first visit, we did not detect gingival swelling, bleeding, or pus discharge (Figure 5).

We conducted a periodontal examination and assessed dental radiographs, photographs, and the presence of periodontal bacteria in the subgingival area by qPCR. At the time of examination, we diagnosed the patient with generalized aggressive periodontitis according to the 1999 classification of periodontal diseases and conditions; however, at the time of this study, the diagnosis was revised to generalized stage III, grade C periodontitis based on the updated classification guidelines [5].

**Figure 6:**
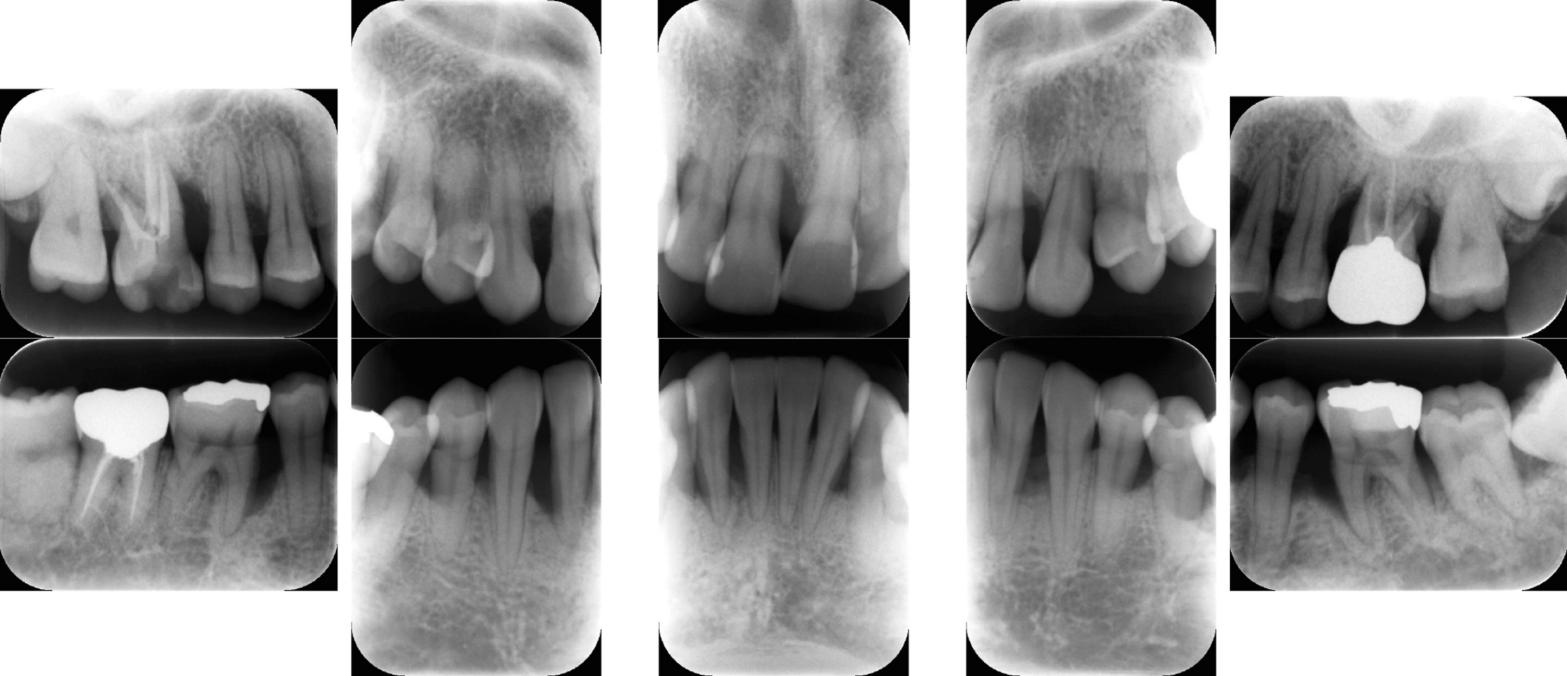
Clinical radiographs of a patient diagnosed with generalized stage III, grade C periodontitis (Case 2). Radiographs at baseline show severe bone loss in multiple sites.

**Figure 7:**
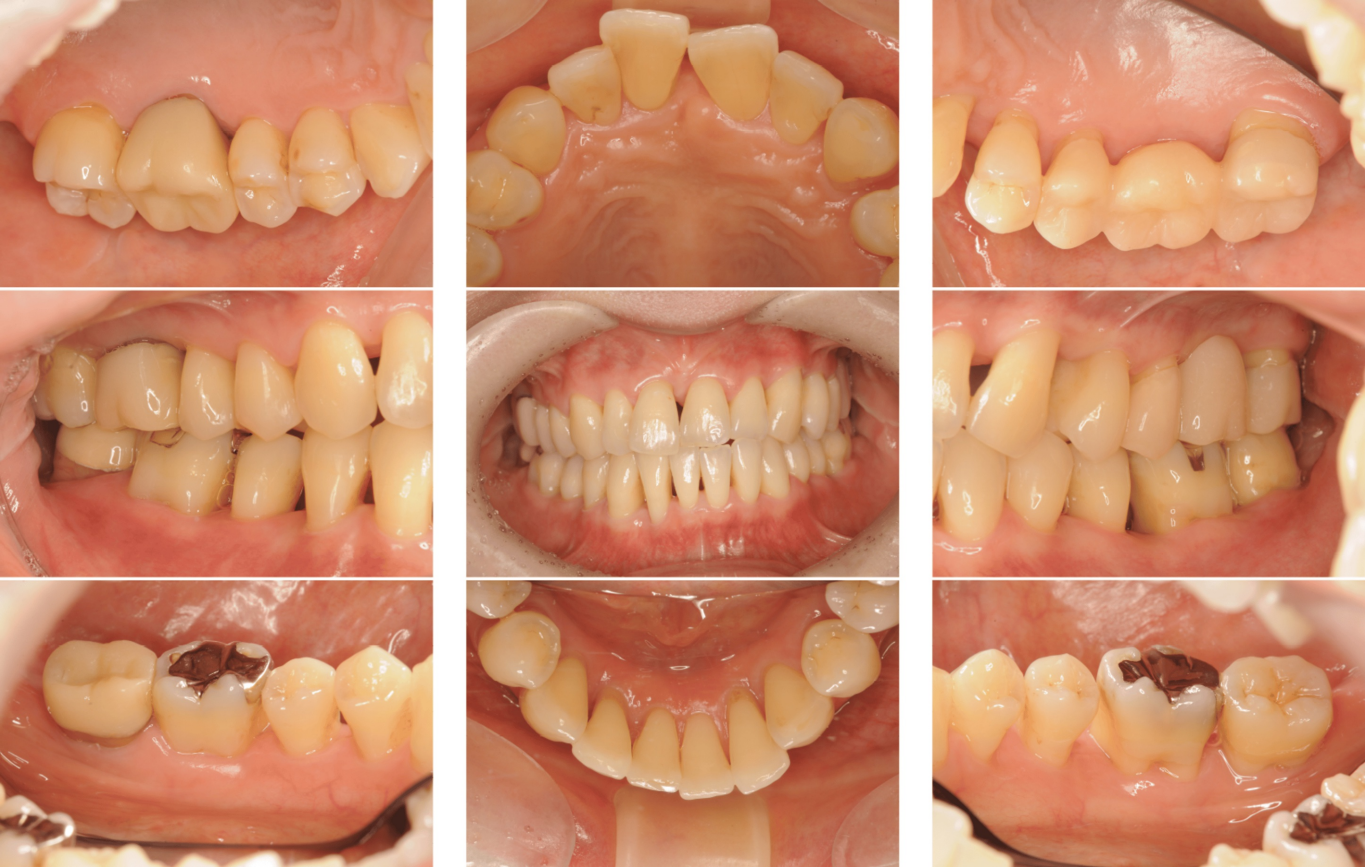
Post-treatment clinical photographs of a patient with generalized stage III, grade C periodontitis (Case 2). Clinical improvements, including reduced inflammation and improved periodontal condition, were observed after FMD and sitafloxacin. FMD: full-mouth disinfection

**Figure 8:**
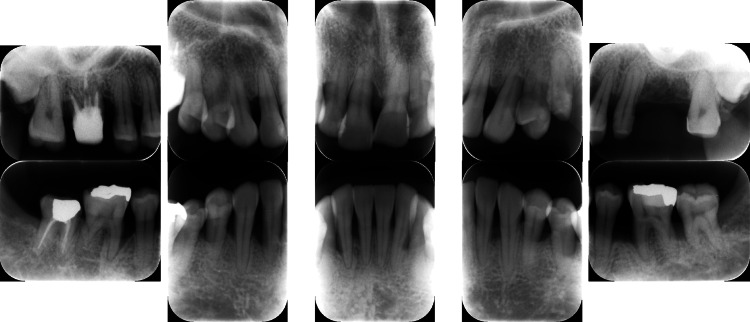
Post-treatment radiographs of a patient with generalized stage III, grade C periodontitis (Case 2). Bone regeneration in vertical defects was observed after FMD and sitafloxacin therapy. FMD: full-mouth disinfection

Treatment was initiated with instructions on oral hygiene and subsequent FMD; debridement of the upper and lower dentition was completed within 2 days, and sitafloxacin was administered from days 1 to 7. At the 6-month follow-up, the O’Leary plaque control record showed a reduction from 39% to 10%. Additionally, other clinical parameters showed substantial improvements (Table 1). In addition, dental radiographs showed no progression of alveolar bone resorption and findings of bone regeneration in the vertical defects in both jaws. Figure 6 shows the lamina dura with a blurry appearance due to inflammatory bone destruction, which later became more distinct as shown in Figures 7-8.

This procedure led to decreased levels of red complex bacteria and increased clinical benefits (Table 3).

**Table 3:** Changes in red complex bacteria counts before and after the FMD and STFX protocol FMD: full-mouth disinfection; STFX: sitafloxacin

	Case 2		Case 3		Case 4	
Subgingival bacteria	Baseline	FMD and STFX	Baseline	FMD and STFX	Baseline	FMD and STFX
Total bacteria	4,600,000	14,000	310,000	1100	1,700,000	300,000
Porphyromonas gingivalis	6900	<10	<10	<10	5000	<10
Tannerella forsythia	680,000	<10	7800	<10	230,000	8900
Treponema denticola	25,000	<10	1700	<10	15,000	<10

Case 3

**Figure 9:**
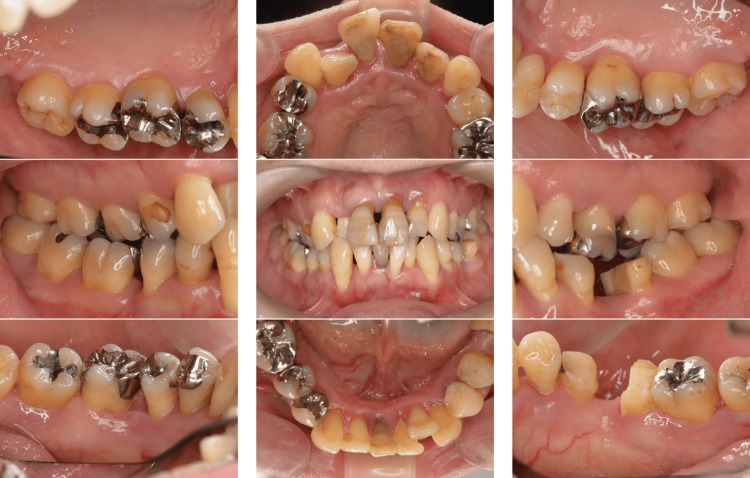
Baseline clinical photographs of a patient with generalized stage III, grade C periodontitis (Case 3). Periodontal inflammation and deep probing pocket depths were observed in multiple sites.

**Figure 10:**
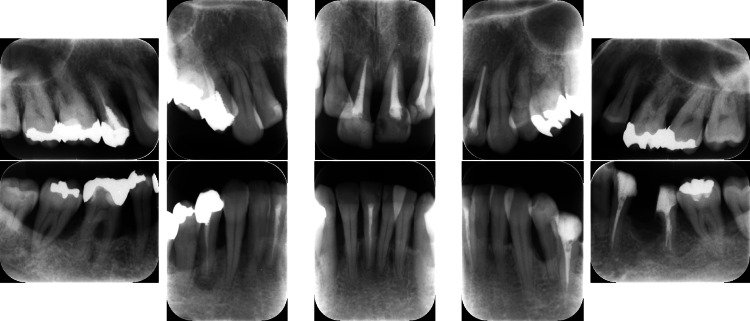
Baseline radiographs of a patient with generalized stage III, grade C periodontitis (Case 3). Severe alveolar bone resorption was noted, particularly in the posterior regions.

A 42-year-old Japanese woman with severe periodontitis was referred to our department for treatment (generalized stage III, grade C periodontitis; Figures 9-10).

**Figure 11:**
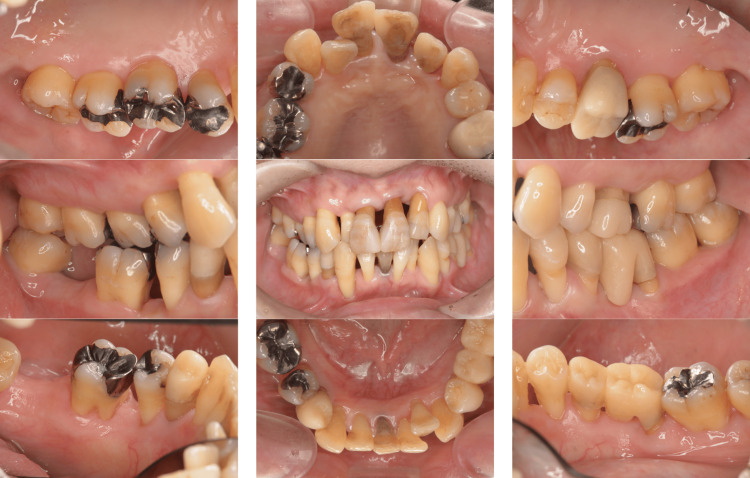
Post-treatment clinical photographs of a patient with generalized stage III, grade C periodontitis (Case 3). Significant clinical improvements were observed, including reduced inflammation and shallower probing depths.

**Figure 12:**
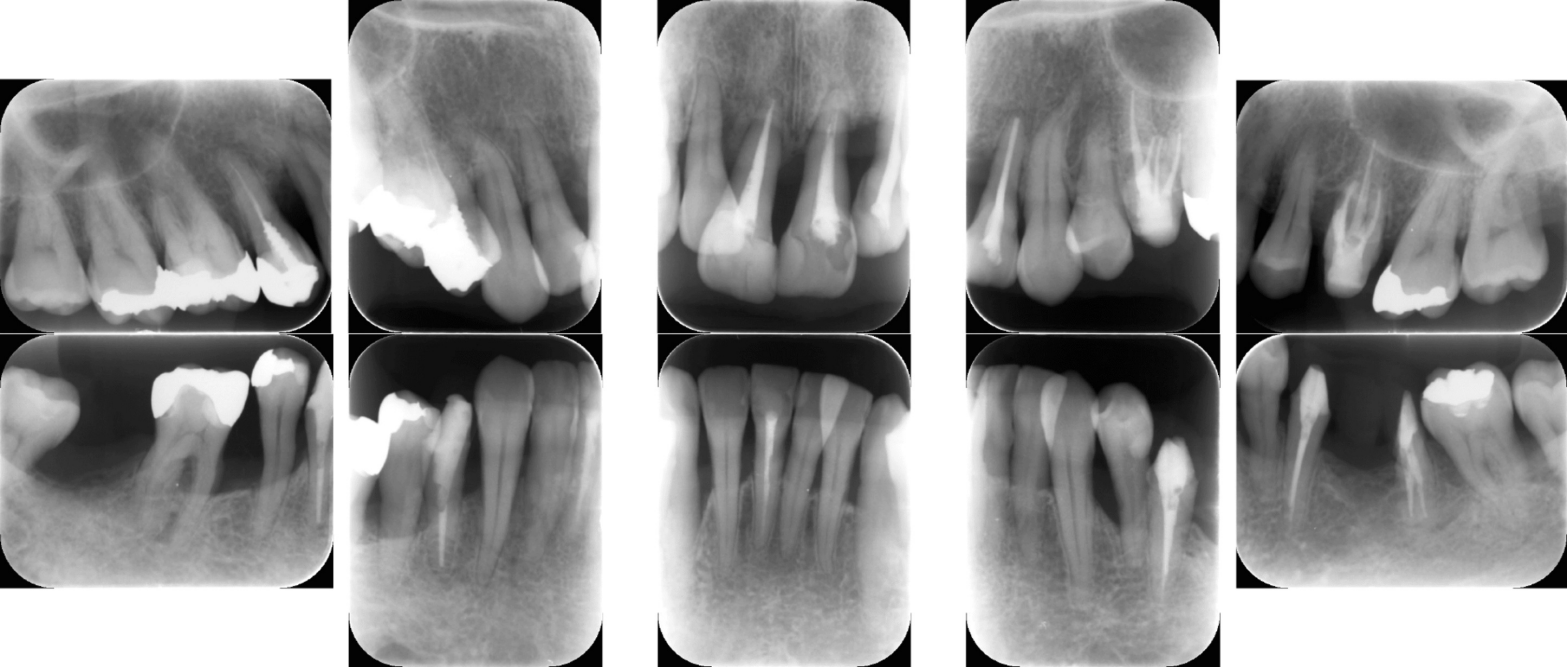
Post-treatment radiographs of a patient with generalized stage III, grade C periodontitis (Case 3). Bone regeneration was evident in the vertical defects on radiographs taken after the completion of FMD and a one-week course of sitafloxacin. FMD: full-mouth disinfection

Similar to the patient in Case 2, the patient in Case 3 had no systemic disease; thus, the same treatment (FMD and sitafloxacin) was administered with good results (Figures 11-12).

At the 3-month follow-up, all clinical parameters exhibited considerable progress. Furthermore, radiographic bone fill was observed at the disease sites after FMD and sitafloxacin treatment (Figure 12). Additionally, PISA values substantially decreased from 2706.3 mm^2^ to 164.8 mm^2^ (Table 1). The reduction in the levels of red complex bacteria was thus clinically advantageous (Table 3).

Case 4

**Figure 13:**
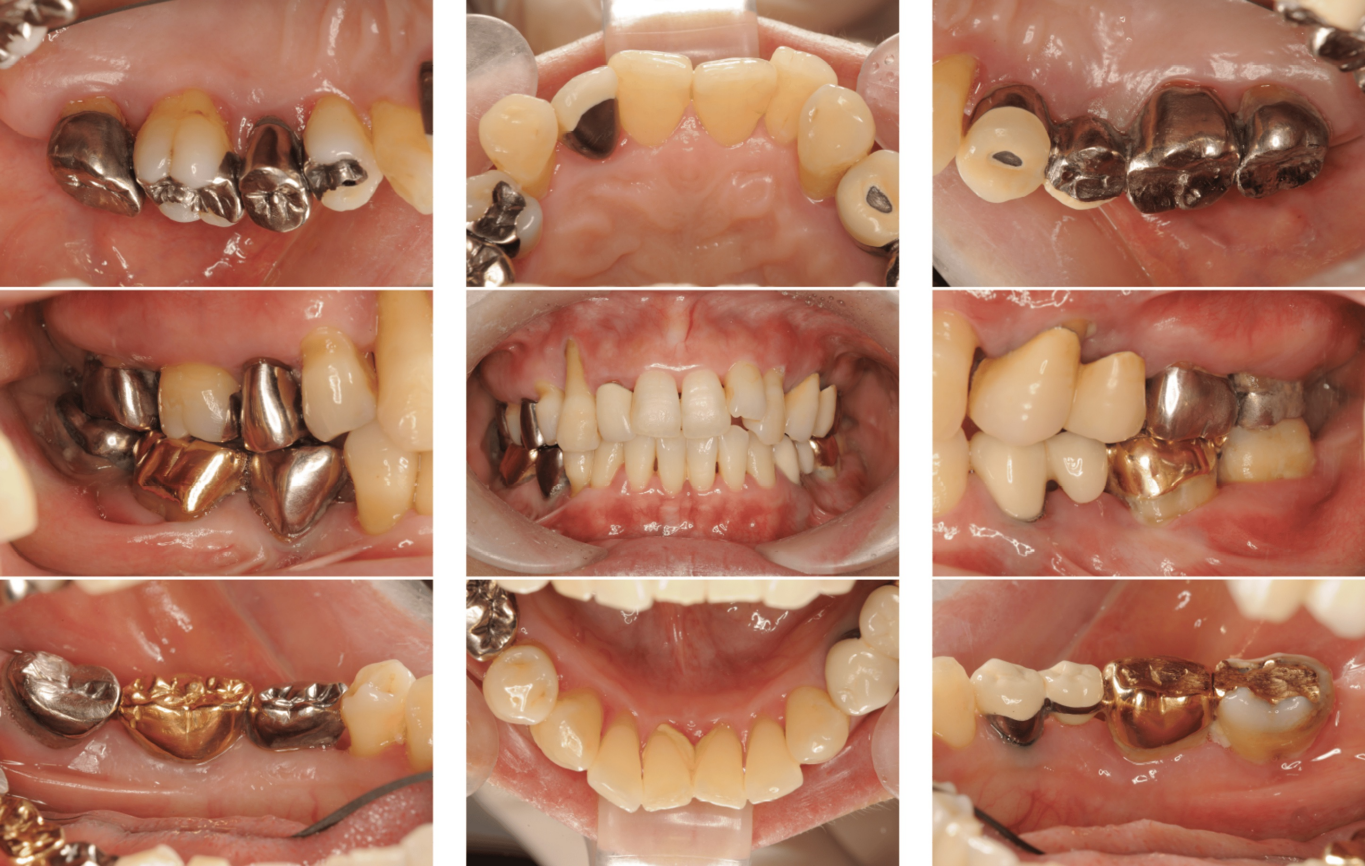
Baseline clinical photographs of a patient with generalized stage IV, grade C periodontitis (Case 4). Severe periodontal destruction, mobility, and deep pockets were noted throughout the dentition.

**Figure 14:**
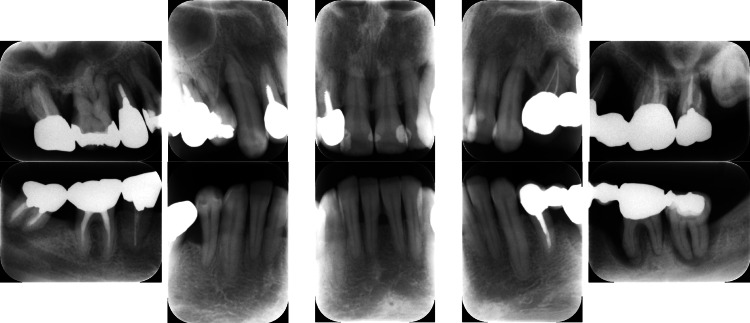
Baseline radiographs of a patient with generalized stage IV, grade C periodontitis (Case 4). Extensive horizontal and vertical bone loss was observed, with furcation involvement in molars.

A 47-year-old Japanese woman was referred to our department for examination and treatment of severe periodontitis, and the patient had no remarkable medical history. A diagnosis of generalized stage IV, grade C periodontitis was established after a thorough examination of her clinical oral health, which included dental photographs, radiographs, and qPCR for the presence of red complex bacteria (Figures 13-14).

The FMD and sitafloxacin protocol showed good performance.

**Figure 15:**
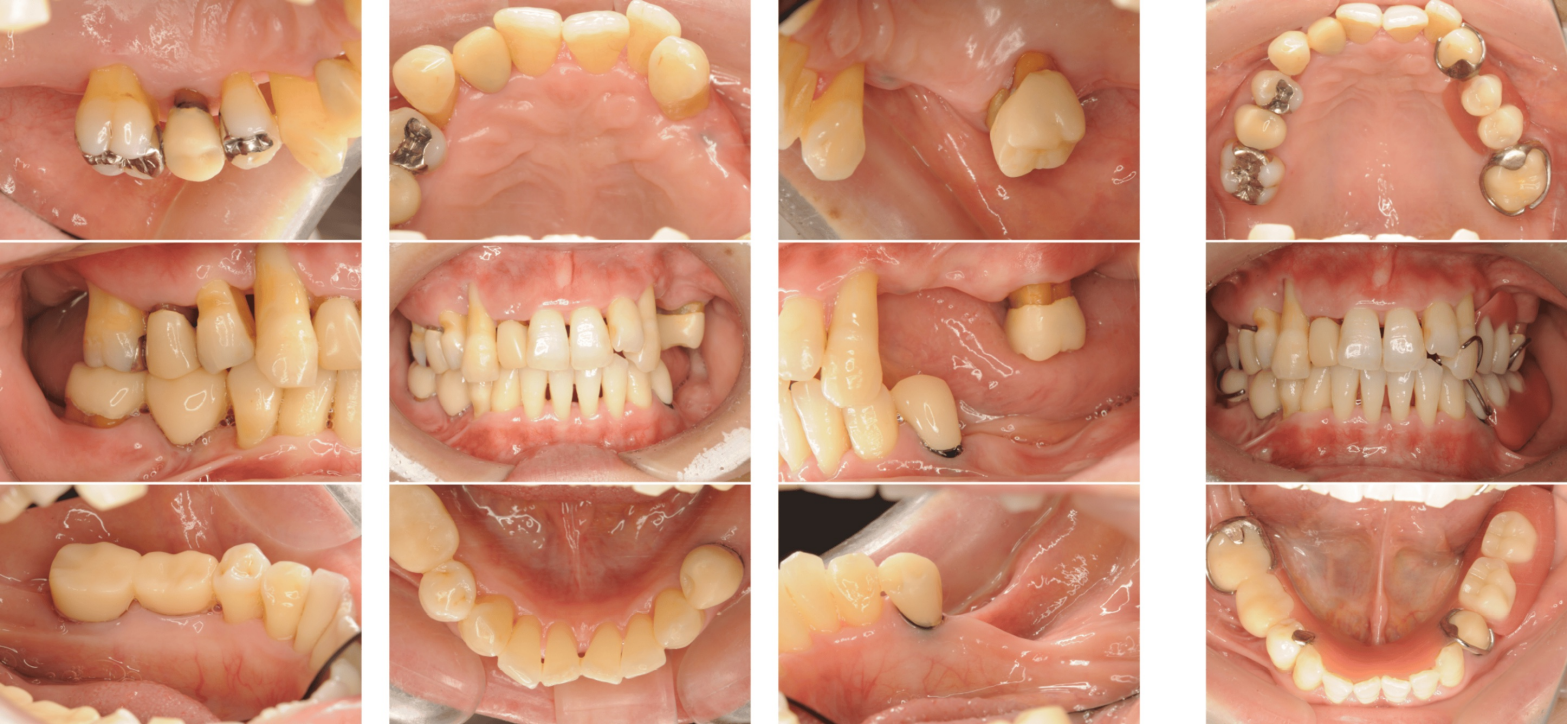
Post-treatment clinical photographs of a patient with generalized stage IV, grade C periodontitis (Case 4). Following FMD and sitafloxacin therapy, clinical improvements were observed, including reduced inflammation and increased soft tissue stability. FMD: full-mouth disinfection

**Figure 16:**
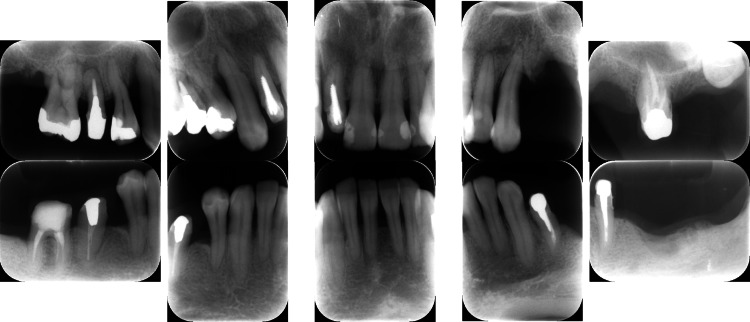
Post-treatment radiographs of a patient with generalized stage IV, grade C periodontitis (Case 4). Radiographic findings indicated bone regeneration in localized vertical defects.

At the 1-month follow-up, as in Cases 2 and 3, the periodontal clinical parameters had improved (Figures 15-16, Table 1).

The decrease in the levels of red complex bacteria was thus clinically advantageous.

## Discussion

This study was undertaken to explore the potential effectiveness of FMD and sitafloxacin as a treatment protocol for severe periodontal diseases with high bacterial levels. Our results indicated that clinical parameters improved drastically after treatment with FMD using systemic sitafloxacin.

A single FMD session can considerably reduce the periodontal bacterial load of the oral cavity. Additionally, FMD reduces the possibility of reinfection from untreated periodontal pockets. While its clinical effects have been widely studied [9], the synergistic effect of combining FMD with antibiotics or chlorhexidine remains controversial. Some studies have shown superior clinical effects of this combination [10], whereas others showed effects comparable to those of traditional SRP [11]. Case 1, which exhibited a high level of subgingival periodontal pathogens, demonstrates that quadrant-based conventional SRP may not produce the expected clinical effect. Thus, it is important to consider an appropriate protocol for generalized stage III and IV, grade C periodontitis with high bacterial levels. Specifically, the treatment should comprise FMD and adjunctive antimicrobial therapy with a limited volume and duration of treatment because bacterial factors are suspected to be more strongly related to periodontal disease than to host or environmental factors. The systemic effects of FMD, such as bacteremia, fever, and a temporary increase in inflammatory cytokine levels, are significant [3]. The clinical parameters of our patients with severe periodontitis improved substantially after treatment with FMD and sitafloxacin. However, according to the original protocol, FMD must be completed within 24 h [12]. A previous study showed that an effective azithromycin concentration could be maintained if SRP was completed within 1 week. Azithromycin has a long half-life, high tissue penetration, more potency than other macrolides against Gram-negative bacteria, and a higher concentration in infected tissues than in non-infected areas. Recent research has shown the successful use of azithromycin in reducing high body temperature following full-mouth SRP [13]. Therefore, the clinical efficacy of full-mouth SRP completed within 1 week with antimicrobial treatment would be comparable to full-mouth SRP termination within 24 h. Furthermore, performing full-mouth subgingival SRP within 1 day can be burdening for patients and frequently induces fever [12]. Hence, we intended to finish SRP in approximately 1 week while monitoring the patient’s physical state and treatment response. Patients with severe periodontal disease present with deep periodontal pockets and the debridement of each tooth surface takes longer than in patients without deep pockets. Moreover, the debridement of several teeth requires sustained concentration from the surgeon. Thus, as an alternative to the original 24-h FMD protocol and conventional SRP over 1-3 months, we suggest a 1-week FMD protocol to minimize physical and mental fatigue and prevent bacterial reinfection from the incompletely debrided site to the completely debrided site. Most patients in this study were treated with the following protocol: On day 1, approximately 14 mandibular teeth were debrided, followed by 14 maxillary teeth on day 2. Oral sitafloxacin was administered 1 h before surgery and continued until day 7. Additionally, antimicrobial medication should be administered immediately before or as soon as possible after debridement, which should preferably be completed within 1 week. Regarding the combination of antimicrobial agents with FMD, the most common antimicrobial agents are amoxicillin and metronidazole [14] or azithromycin [4]. A previous meta-analysis reported the therapeutic effects of a combination of amoxicillin and metronidazole as an adjuvant to SRP [15]. The full-mouth weighted mean reduction in the PPD was 1.41 mm, whereas the gain in clinical attachment level was 0.94 mm. The clinical effects of nonsurgical periodontal therapy can be enhanced by systemic antimicrobial therapy using a combination of amoxicillin and metronidazole.

Given the recent emergence of resistant bacteria (in the body and the environment), there is a worldwide movement regarding the appropriate administration of antimicrobial agents in the oral cavity. In this context, periodontitis treatment involving antimicrobial agents should be the last resort to prevent bacteremia in patients with high systemic risk or difficult mechanical treatment of periodontitis [16]. However, in cases of severe periodontitis, such as in Case 1, there is a high risk of reinfection of periodontal keystone bacteria from untreated to treated sites after conventional periodontal therapy. Since a clinical response to basic treatment was not observed in Case 1, we performed re-SRP as FMD combined with short-term sitafloxacin administration, which resulted in positive outcomes.

A standard procedure and a particular antibiotic dosage for generalized III and IV, grade C periodontitis with high bacterial levels treatment have not yet been determined. Administering antibiotics immediately after mechanical debridement leads to the highest reduction in PPD and improvement in clinical attachment levels in chronic and aggressive periodontitis compared with SRP alone and posterior antibiotic administration [17]. As such, in the four cases described here, adjunctive sitafloxacin was prescribed, resulting in a considerable reduction in red complex bacteria in active periodontal pockets. In addition, we chose a 7-day antibiotic regimen to avoid the emergence of antibiotic resistance and negative effects. According to earlier research on antibiotic resistance, a single course of systemic antibiotics combined with mechanical debridement produced transient bacterial resistance that swiftly vanished after the treatment ceased [18].

No antimicrobial agents were subsequently used for acute periodontitis in all cases of FMD and sitafloxacin administration. Although reliable data are still required to support a microbiological diagnosis prior to treatment selection, there are compelling reasons to be careful while prescribing antimicrobial agents, particularly to minimize bacterial resistance. However, we believe that this protocol, including active and supportive periodontal therapy, should be used once in a patient’s lifetime to avoid excessive cementum grinding and to reduce antimicrobial agent usage for periodontitis to zero in the future.

Recent reports suggest that subgingival air-polishing is more useful than conventional mechanical debridement in supportive periodontal therapy [19]. Similarly, a recent randomized controlled trial revealed that FMD (comprising 0.2-1% chlorhexidine with no antimicrobial medication) and FMD combined with a subgingival erythritol air-polishing were clinically useful in stage III/IV periodontitis compared with conventional quadrant-wise SRP [20]. According to these reports, even in stage III and IV, grade C periodontitis, we may need to shift to these new adjuvant therapies based on the full-mouth concept while avoiding the overuse of antimicrobial agents and high-concentration antiseptics to prevent bacterial resistance and systemic side effects. Conversely, the proposed method should still be useful in periodontal disease treatment in patients who are immunocompromised, systemically susceptible to infection, or at risk of developing infective endocarditis.

In this study, we retrospectively evaluated the effectiveness of FMD under oral sitafloxacin in treating severe periodontitis in which many red complexes were detected, and the clinical parameters and quantitative evaluation of red complexes confirmed the usefulness of this protocol. When antimicrobial agents are used, the emergence of resistant strains due to overuse should be considered. The clinical efficacy of this protocol can be expected in stage III/IV severe periodontitis and in severe periodontitis, in which bacterial factors are considered to play a significant role.

This study’s main limitation is the small sample size; therefore, a randomized controlled study with a large number of patients obtainable through collaborative research is needed to confirm our results.

## Conclusions

This case series suggests that FMD combined with STFX can be an effective treatment for severe periodontitis cases with high bacterial levels, offering advantages over conventional SRP in terms of clinical outcomes and bacterial reduction. However, larger randomized controlled trials are needed to confirm these findings and establish clear guidelines for their use. Future research should focus on optimizing the protocol, perhaps by exploring the use of locally delivered antibiotics or alternative antimicrobial agents to minimize systemic exposure. Additionally, comparative studies with other emerging treatments, such as erythritol powder air-polishing, could help determine the most effective approaches for managing severe periodontitis and guide clinicians in selecting the best treatment options for their patients.
